# Virtual Reality–Based Mirror Guidance for Indirect Vision Training in Minimally Invasive Endodontic Access Cavity Preparation Among Dental Students: Randomized Controlled Crossover Trial

**DOI:** 10.2196/100884

**Published:** 2026-10-01

**Authors:** Unthitar Suranin, Metasit Getrak, Siriwan Suebnukarn, Peter Haddawy, Myat Su Yin, Rene Weller, Panuroot Aguilar, Kamon Budsaba, Andre Mühlenbrock, Gabriel Zachmann

**Affiliations:** 1 Division of Endodontics, Faculty of Dentistry, Thammasat University Pathum Thani Thailand; 2 Computer Graphics and Virtual Reality, University of Bremen Bremen Germany; 3 Faculty of Information and Communication Technology, Mahidol University Nakhon Pathom Thailand; 4 Bremen Spatial Cognition Center, University of Bremen Bremen Germany; 5 Department of Tropical Hygiene, Faculty of Tropical Medicine, Mahidol University Bangkok Thailand; 6 Department of Mathematics and Statistics, Thammasat University Pathum Thani Thailand

**Keywords:** dental education, endodontics, indirect vision, minimally invasive dentistry, mirror guidance, simulation training, virtual reality, VR

## Abstract

**Background:**

Minimally invasive endodontics requires precise visualization to preserve tooth structure, often relying on indirect vision with a dental mirror—a challenging skill for dental students. Although virtual reality (VR) simulators provide immersive environments for dental training, most existing systems primarily focus on procedural simulation and haptic feedback, with limited capabilities for real-time mirror positioning and indirect visual guidance.

**Objective:**

The aim of this study was to evaluate verbal, visual, and haptic VR-based mirror guidance for indirect vision during minimally invasive endodontic access cavity preparation.

**Methods:**

Eligible fourth-year preclinical dental students were recruited through institutional email invitations and public announcements. A randomized controlled crossover trial was conducted with 30 participants, who completed all VR sessions in a simulation laboratory under direct investigator supervision to ensure standardized implementation of the training protocol. Participants performed simulated maxillary first molar access cavity preparations under 4 VR mirror guidance conditions—verbal guidance, visual guidance, haptic guidance, and no guidance—following a randomized 4 × 4 Latin square sequence with a 48-hour washout period between sessions. The primary outcome was outcome error score. Exploratory secondary outcomes included operating area visibility, operating area obstruction, operator posture error, task completion time, and Virtual Reality System Usability Questionnaire score. Outcomes were analyzed using linear mixed-effects models accounting for the crossover design. Estimated marginal mean differences (MDs) with 95% CIs and partial eta-squared (ηp^2^) effect sizes were reported, with Bonferroni-adjusted pairwise comparisons used to account for multiple comparisons.

**Results:**

Guidance modality significantly affected the primary outcome, outcome error score (*F*_3,84_=20.34; *P*<.001; ηp^2^=0.421). Compared with no guidance, outcome error scores were significantly lower with verbal (MD −0.50, 95% CI −0.76 to −0.24), visual (MD −0.68, 95% CI −0.94 to −0.42), and haptic guidance (MD −0.63, 95% CI −0.89 to −0.37; all adjusted *P*<.001). Guidance modality also significantly affected operating area visibility (*F*_3,84_=12.31; *P*<.001; ηp^2^=0.305). Compared with no guidance, visibility duration was significantly greater with verbal (MD 37.79, 95% CI 11.15-64.43 seconds; adjusted *P*=.001), visual (MD 59.15, 95% CI 32.51-85.79 seconds; adjusted *P*<.001), and haptic guidance (MD 31.52, 95% CI 4.90-58.15 seconds; adjusted *P*=.01). No significant differences were detected for operating area obstruction, operator posture error, task completion time, or usability.

**Conclusions:**

VR-based mirror guidance improved the outcome error score and increased operating area visibility during minimally invasive endodontic access cavity preparation. Verbal, visual, and haptic guidance resulted in fewer outcome errors and greater operating area visibility than no guidance, while no significant differences were detected in other performance measures or usability. These findings demonstrate the potential of diverse feedback modalities to provide effective guidance for indirect vision and inform future development of adaptive VR training systems using 1 or more modalities to suit learner needs.

**Trial Registration:**

Thai Clinical Trials Registry TCTR20260126008; https://www.thaiclinicaltrials.org/show/TCTR20260126008

## Introduction

### Background

Virtual reality (VR) has become an increasingly prominent tool in health professions education, offering immersive simulation, standardized training environments, and objective performance assessment [1]. Simulation-based education has been shown to enhance psychomotor skill acquisition, facilitate deliberate practice, and reduce reliance on variable instructor-dependent feedback [2]. By enabling repetitive, risk-free practice with integrated performance metrics, VR platforms provide opportunities to enhance technical training while supporting evidence-based educational design [3].

In dental education, VR simulators have been introduced to support psychomotor skill training. These systems allow learners to practice cavity preparation, spatial hand-eye coordination, and instrument control within a controlled digital environment. Previous studies have demonstrated that VR-based dental simulators can improve technical precision, increase learner confidence, and provide reliable performance tracking [4,5]. Recent systematic reviews and meta-analyses have further confirmed the effectiveness of VR simulation in dental education, demonstrating positive effects on knowledge acquisition, technical performance, and learner satisfaction across various disciplines [6]. The application of VR and AR technologies has also expanded into operative dentistry, endodontics, and oral and maxillofacial surgery education, reflecting their increasing role in supporting clinical skills training and competency development [7-9]. Beyond procedural replication, modern VR platforms increasingly incorporate real-time feedback mechanisms, reflecting the growing recognition that structured augmented feedback is central to effective motor skill development [10,11]. Evidence from immersive learning research further suggests that virtual environments can enhance psychomotor skill acquisition, spatial understanding, and learner engagement through realistic and interactive training experiences [12].

Minimally invasive dentistry, particularly minimally invasive endodontic access cavity preparation, requires a high level of precision and spatial awareness [13]. Contemporary endodontic philosophy emphasizes preservation of pericervical dentin and conservation of coronal tooth structure to maintain fracture resistance and long-term tooth survival [14]. However, reducing access cavity size must be carefully balanced against adequate visualization of the pulp chamber and canal orifices. Insufficient access or compromised visualization may increase the risk of missed canals, iatrogenic errors, and procedural complications [15,16]. Achieving this balance depends heavily on accurate indirect vision using a dental mirror. For novice learners, maintaining appropriate mirror orientation while simultaneously controlling bur movement presents a substantial cognitive and psychomotor challenge.

Despite the importance of indirect vision control, structured training of mirror orientation remains limited. Traditional instruction relies primarily on instructor observation and verbal correction, which may vary in timing, frequency, and specificity. While existing VR dental simulators focus largely on bur position, cavity form, and volumetric preparation metrics, few systems incorporate dedicated mirror alignment guidance as an explicit instructional component [5,17]. Given the critical role of visualization in minimally invasive access cavity preparation, the absence of structured mirror feedback represents a meaningful educational gap.

Effective psychomotor training depends not only on practice but also on well-designed augmented feedback. Motor learning theory emphasizes the importance of knowledge of performance feedback in supporting early-stage skill acquisition and refining movement patterns [18]. At the same time, Cognitive Load Theory posits that instructional support must be carefully calibrated to minimize extraneous cognitive load and optimize working memory resources during complex tasks [19]. Multimedia Learning Theory further suggests that feedback modalities should be integrated coherently to avoid competing sensory demands [20]. Therefore, the integration of structured, multimodal mirror guidance within VR environments represents a promising but underexplored strategy for enhancing indirect vision training in minimally invasive dentistry.

To address this need, a mirror positioning guidance module has been developed as an extension of the previously validated VR-based dental training platform [17]. The current iteration integrates 3 real-time feedback modalities—verbal, visual, and haptic—to support learners in maintaining appropriate mirror orientation during minimally invasive operation. The educational impact of different feedback modalities for VR-based mirror guidance has not been systematically investigated. Understanding how verbal, visual, and haptic feedback influence mirror orientation performance and learner experience is essential for evidence-based design of VR-supported preclinical dental education.

### Study Objectives

The aim of this study was to evaluate verbal, visual, and haptic VR-based mirror guidance for indirect vision during minimally invasive endodontic access cavity preparation. The primary outcome was outcome error score, while operating area visibility, operating area obstruction, operator posture error, task completion time, and usability were evaluated as exploratory secondary outcomes. We hypothesized that the different VR-based mirror guidance modalities would produce different effects on indirect vision performance, with guided conditions expected to reduce outcome error score compared with the no-guidance control condition.

## Methods

### Ethical Considerations

This study was approved by the Human Research Ethics Committee of Thammasat University (approval number COA No. 003/2569) on January 22, 2026. The study was conducted in accordance with the ethical principles of the Declaration of Helsinki. The trial was prospectively registered with the Thai Clinical Trials Registry (TCTR20260126008) on January 26, 2026, before enrollment of the first participant on July 25, 2026. All participants provided written informed consent before participation. Participation was voluntary, and participants were informed of their right to withdraw from the study at any time without academic consequences. Participant data were anonymized before analysis and securely stored to ensure confidentiality. No personally identifiable information or identifiable participant images are included in this manuscript. Participants received financial compensation of 50 Thai baht (THB; approximately US $1.50) for each experimental session, consistent with the institutional rate for student research assistants. As each participant completed 4 experimental sessions, the total compensation was 200 THB (approximately US $6) per participant. The study was designed, conducted, and reported in accordance with the CONSORT (Consolidated Standards of Reporting Trials) 2025 statement and the CONSORT-EHEALTH (Consolidated Standards of Reporting Trials of Electronic and Mobile Health) guidelines [21,22]. The CONSORT checklist and the trial protocol are available in Multimedia Appendices 1 and 2.

### Overview

A randomized crossover trial was conducted to evaluate the effectiveness and usability of different VR-based mirror guidance modalities for improving indirect vision during minimally invasive endodontic access cavity preparation.

### VR-Based Mirror Guidance

The VR dental simulator was developed using Unreal Engine (UE) 4.27.2. Stereo visualization was provided through an HTC Vive Pro Eye head-mounted display (resolution 2880 × 1600) integrated with eye-tracking sensors using the SteamVR and SRanipal Unreal plugins. A virtual dental handpiece and mouth mirror were manipulated using 2 Geomagic Touch haptic devices (3D Systems), providing 6 degrees of freedom and force feedback to simulate tactile interaction with the virtual tooth. Realistic drilling sounds and visual rendering were incorporated to enhance immersion (Figure 1). The source code for the VR-based mirror guidance system and demonstration materials, including screenshots and video recordings, are provided in Multimedia Appendices 3 and 4.

**Figure 1:**
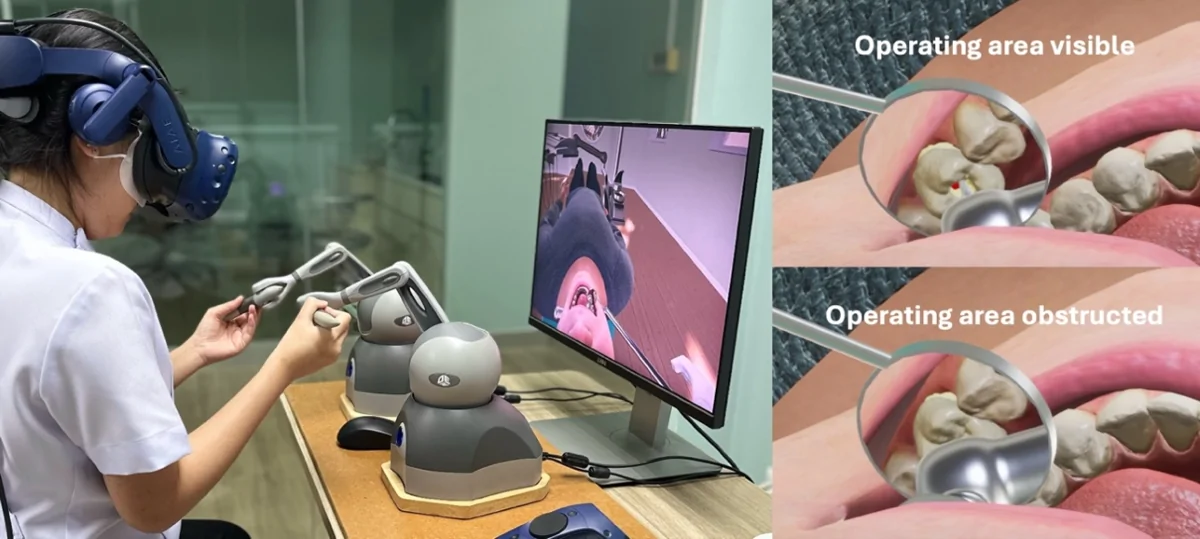
Virtual reality (VR) dental simulator and examples of operating area visibility assessment during minimally invasive endodontic access cavity preparation using indirect vision. The simulator used in this randomized controlled crossover trial allowed fourth-year preclinical dental students to manipulate a virtual dental handpiece and mirror using a head-mounted display and haptic devices. Representative images demonstrate conditions in which the operative field (including the bur tip and the occlusal cutting area) was either visible or obstructed during access cavity preparation.

A virtual patient model was created using the MetaHuman framework and integrated into the UE environment. The left maxillary first molar (tooth 26) was replaced with a custom tooth model reconstructed from microcomputed tomography (micro-CT) scans of human teeth to provide anatomically realistic internal structures. The model was implemented using UE’s Procedural Mesh Component and dynamically rendered using a metaballs-based surface reconstruction approach. Haptic interactions during drilling were simulated using a sphere-packing representation of enamel, dentin, and pulp tissues, with force and friction parameters calibrated in consultation with an expert dentist.

### Mirror Correction Algorithm

#### Overview

The mirror correction algorithm was developed to detect suboptimal mirror positioning and automatically trigger appropriate VR guidance. All mirror guidance modalities (verbal, visual, and haptic) were designed to assist the user in adjusting the dental mirror so that the tooth remained visible when the dental handpiece contacted the tooth. The guidance system operated under 3 conditions that determined when corrective feedback was applied.

#### Condition A: Mirror Position Outside the Optimal Area

Guidance was triggered when the mirror position was outside a predefined optimal region where the mirror was expected to be positioned. As illustrated in Figure 2, the optimal region was defined as a conical space characterized by 5 parameters: origin point (o), inner radius (r_1_), outer radius (r_2_), cone direction (d), and cone angle (θ).

**Figure 2:**
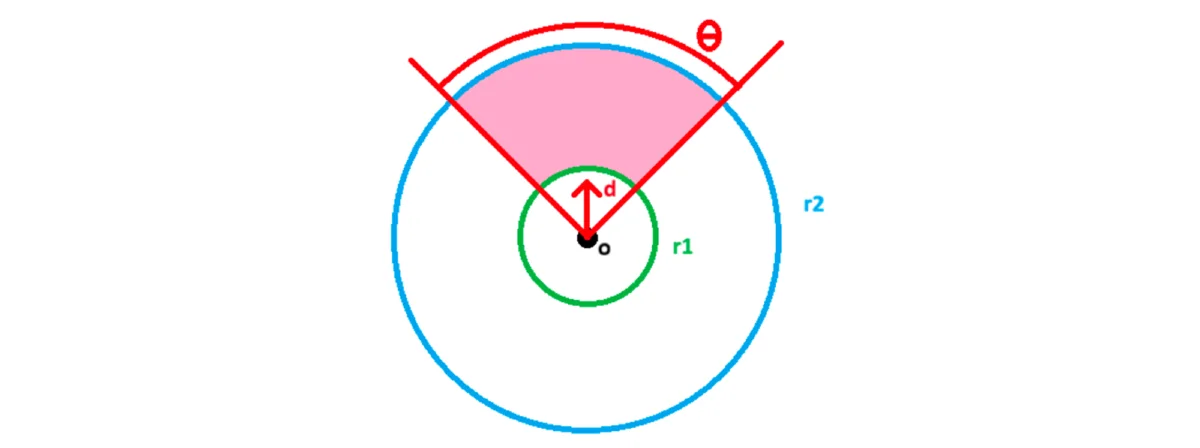
Visualization of the mirror correction algorithm used in the virtual reality–based mirror guidance system for indirect vision training during minimally invasive endodontic access cavity preparation. The optimal mirror positioning region is modeled as a conical space defined by the origin point (o), inner radius (r1), outer radius (r2), cone direction (d), and cone angle (θ). Mirror positions located within this predefined region were considered acceptable for achieving appropriate reflection of the operating area during the randomized controlled crossover trial.

To determine whether the mirror was within the optimal region, the following procedure was applied:

- Let p represent the mirror position in 3D space.
- Compute the unit vector d_p_ from o to p.
- Calculate the angle (θ_p_ between d and d_p_.

If θ_p_ < θ/2 and the distance between p and o lies between r_1_ and r_2_, the mirror is considered inside the optimal region.

#### Condition B: Incorrect Mirror Orientation

When the mirror was positioned within the optimal region but oriented incorrectly, the reflected image did not allow the tooth to be seen by the user. Mirror orientation was evaluated based on the user’s head and eye positions and the mirror’s reflection geometry.

The following variables were used: user head position (p_h_), left eye position (p_le_), right eye position (p_re_), mirror position (p_m_), mirror normal (n_m_), tooth position (p_t_), and a base angular threshold (θ_th_).

The orientation algorithm consisted of the following steps:

- Compute the distance (d) between p_t_ and p_m_.
- Adjust the angular threshold (θ′_th_ based on d, such that the allowable angle decreases as the mirror-tooth distance increases.
- Calculate the reflection vector (r) using the tooth position (p_t_) as the source and the mirror position (p_m_) and normal (n_m_) as the reflection parameters.
- Compute normalized target vectors from the mirror to the head (t_h_), left eye (t_le_), and right eye (t_re_).
- Calculate the angle between r and each target vector.
- If the smallest angle exceeded θ′_th_, the tooth was considered not visible in the mirror.

#### Condition C: Operating Area Obstructed by the Handpiece

Even when the mirror was correctly positioned and oriented, the operating area could still be obstructed by the dental handpiece. To detect occlusion, the following variables were considered: tooth tip position (p_t_), handpiece head position (p_b_), handpiece head radius (r_b_), and mirror position (p_m_).

The occlusion detection algorithm was implemented as follows:

- Construct a line (l) from the tooth tip (p_t_) to the mirror (p_m_).
- Compute the distance (d_lb_) between the handpiece head (p_b_) and the closest point on line (l).
- If d_lb_ > r_b_, the view was unobstructed; otherwise, the view was considered obstructed.

When occlusion occurred, a suggested mirror position (p′_m_) was computed by identifying tangent lines between the tooth tip and the handpiece head. The suggested mirror position was selected along the tangent line that produced the smallest angular deviation from the original viewing line while maintaining the same distance from the tooth.

#### Verbal Guidance

Verbal guidance was implemented to notify users when mirror adjustment was required during indirect vision tasks. The system assumed that dental students were familiar with basic mirror manipulation and therefore provided brief corrective prompts rather than detailed instructions. When the mirror correction algorithm detected one of the predefined conditions described in the previous section, prerecorded audio cues were triggered as follows:

- Condition A: “move mirror to the mouth.”
- Condition B: “adjust mirror.”
- Condition C: “tool tip is not visible.”

#### Visual Guidance

The visual guidance system incorporated multiple visual cues, including a ghost mirror, a reflection line, and a cone visualization, each activated according to specific mirror positioning conditions (Figure 3). These components were designed to provide spatial information to assist learners in achieving appropriate mirror alignment and indirect visualization. The ghost mirror represented the ideal mirror position and orientation required for proper indirect visualization of the tooth. When displayed, users were instructed to align the real mirror with the ghost mirror. The ghost mirror orientation was computed based on reflection geometry such that the tooth location would appear in the mirror when viewed by the user. The opacity of the ghost mirror was dynamically adjusted according to positional and angular differences between the real mirror and the ideal mirror position, providing continuous visual feedback during alignment. Similar ghost tool visualization has previously been used to support medical skills training [23].

**Figure 3:**
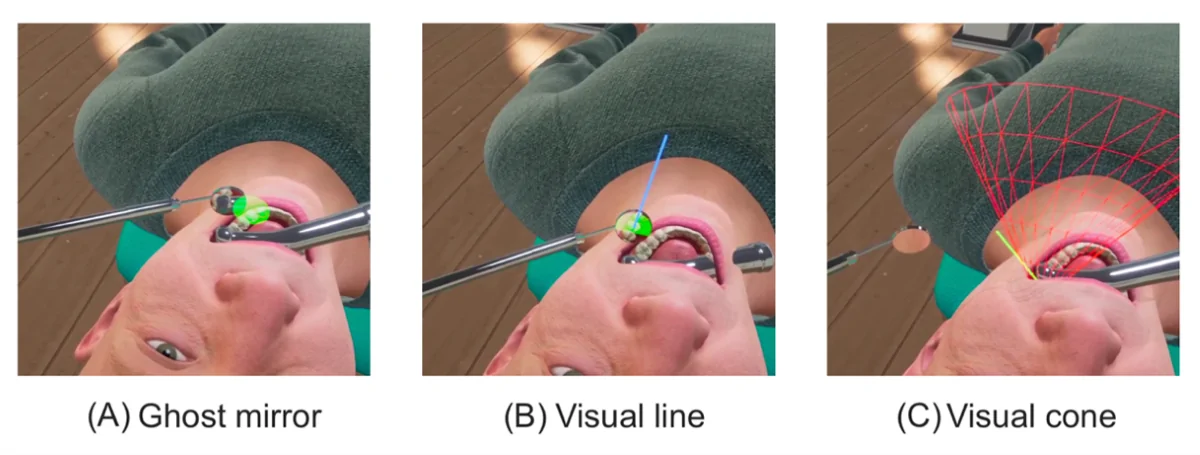
Visual guidance components of the virtual reality–based mirror guidance system. (A) Ghost mirror indicating the ideal mirror position and orientation to achieve indirect visualization. (B) Reflection line representing the reflection vector from the tooth surface to facilitate fine adjustment of mirror orientation. (C) Cone visualization indicating the predefined optimal spatial region for mirror placement during minimally invasive endodontic access cavity preparation.

To assist with fine adjustment of mirror orientation, a reflection line was also displayed. The reflection line represented the reflection vector of the tooth on the mirror surface. When the mirror was oriented correctly, this line pointed toward the user’s head position, indicating that the tooth would be visible in the mirror. The opacity of the reflection line decreased as the reflection vector approached the user’s head, minimizing visual distraction once the correct orientation was achieved.

Finally, when the mirror position moved outside the predefined optimal region, a cone visualization was displayed. This cone indicated the acceptable spatial region for mirror placement and guided the user to reposition the mirror back into the optimal area. Based on the mirror correction conditions described in the previous section, visual guidance was applied as follows:

- Condition A: the cone visualization was displayed to guide the mirror back into the optimal region.
- Condition B: the ghost mirror and reflection line were displayed at the current mirror position to assist with orientation correction.
- Condition C: the ghost mirror was displayed at the suggested mirror position calculated by the algorithm to help restore visibility of the operating area.

#### Haptic Guidance

The haptic guidance strategy integrated corrective force feedback with haptic disturbance to enhance user awareness of inappropriate mirror positioning. Feedback was applied according to the mirror correction conditions described previously.

- Condition A: when the mirror was located outside the optimal region, a direct haptic force was applied to the mirror device, directing the user’s hand toward the optimal area.
- Condition B: when the mirror was positioned within the optimal region but incorrectly oriented, the system referenced the most recent mirror position at which the tooth was successfully visible. Let this position be p, and the current mirror position be p_m_.
- If the distance between p and p_m_ exceeded a predefined threshold (d_th_, a corrective force was applied to guide the mirror from p_m_ toward p.
- If the distance between p and p_m_ was below the threshold but the angular difference exceeded an orientation threshold (θ_th_), haptic noise was applied to the device to indicate that the mirror orientation should be adjusted. Haptic noise has previously been shown to support motor skill learning [24].
- Condition C: when the operating area was obstructed by the handpiece, a corrective force was applied to guide the mirror from its current position toward the suggested mirror position calculated by the algorithm.

### Patient and Public Involvement

Patients and members of the public were not involved in the design, conduct, reporting, or dissemination planning of this trial. The study involved fourth-year preclinical dental students as participants and evaluated a VR-based educational intervention.

### Trial Design

This study was conducted as a randomized 4 × 4 Latin square crossover trial to evaluate the effectiveness and usability of different VR-based mirror guidance modalities, including verbal, visual, and haptic guidance, for improving indirect vision during minimally invasive endodontic access cavity preparation.

### Changes to Trial Protocol

The study protocol was amended to increase the approved sample size, with the amendment approved on July 17, 2026. No changes were made to the study outcomes or intervention protocol.

### Trial Setting

The trial was conducted in the VR simulation laboratory at the Faculty of Dentistry, Thammasat University, Thailand.

### Eligibility Criteria

Fourth-year preclinical dental students who had successfully completed the preclinical endodontics course were eligible to participate. Students with prior experience using a dental VR simulator were excluded.

No additional eligibility criteria were specified for the study site because the trial was conducted at a single simulation laboratory. No eligibility criteria were specified for intervention providers because the assigned guidance was delivered automatically by the VR system. The supervising investigator provided standardized orientation and technical support but did not provide instructional guidance or performance feedback during the experimental tasks.

### Intervention and Comparator

#### Overview

The intervention consisted of real-time VR-based guidance for dental mirror positioning during minimally invasive endodontic access cavity preparation. It was delivered through a VR dental simulation system incorporating a head-mounted display, 2 haptic devices, and real-time performance monitoring.

#### Core Components and Mode of Delivery

The intervention comprised 3 active guidance conditions—verbal, visual, and haptic guidance—and a no-guidance control condition. All guidance was delivered automatically by the VR system during task performance. A mirror positioning correction algorithm continuously monitored the position and orientation of the virtual dental mirror during access cavity preparation. When suboptimal mirror positioning resulted in reduced visibility of the operating area, the system automatically provided real-time guidance to assist the participant in adjusting the mirror to restore appropriate indirect visualization. Verbal guidance provided auditory instructions, visual guidance displayed directional cues within the VR environment, and haptic guidance delivered force-based directional cues through the haptic device controlling the dental mirror. In the no-guidance condition, participants performed the same task using the same VR system without these corrective guidance cues. Guidance in the 3 active conditions was delivered synchronously and was automatically triggered according to the participant’s real-time mirror positioning performance. No instructor-delivered mirror positioning feedback, external communication, web-based interaction, or adaptive educational content beyond the mirror correction algorithm was incorporated.

#### Setting, Equipment, and Intervention Provider

All sessions were conducted individually in a supervised simulation laboratory at Thammasat University Faculty of Dentistry. The VR simulator incorporated a head-mounted display and 2 Geomagic Touch haptic devices, which provided 6-degree-of-freedom positional sensing and 3-degree-of-freedom force feedback for manipulation of the virtual dental handpiece and dental mirror. Before participant enrollment, the VR simulator and mirror guidance modules underwent technical testing to ensure stable system performance, accurate delivery of verbal, visual, and haptic guidance, and reliable recording of performance data. The virtual training environment, instrument settings, and guidance parameters were standardized and remained unchanged throughout the study. Participants did not require personal devices, internet access, or payment to use the system.

The VR system itself delivered the assigned guidance automatically. Before the first experimental session, an investigator provided each participant with standardized face-to-face orientation and instructions on operation of the VR simulator and study procedures. During experimental sessions, an investigator was present to supervise protocol implementation and provide technical support only; no additional instructional coaching, mirror positioning guidance, or performance feedback was provided by the investigator.

#### Exposure and Schedule

Each participant performed a minimally invasive endodontic access cavity preparation on tooth 26 (maxillary left first molar) under all 4 guidance conditions. The order of conditions was determined by the randomized crossover sequence. There was no fixed duration for an experimental session; participants continued the task until access cavity preparation was completed. Consecutive sessions were separated by a 48-hour interval to minimize immediate carryover effects, including fatigue and short-term adaptation to the preceding guidance condition; however, this interval was not intended to eliminate longer-term motor learning or skill acquisition associated with repeated task performance [25]. Participants requiring visual correction were instructed to wear their habitual eyeglasses or contact lenses during all VR sessions to ensure that tasks were performed under their normal corrected visual conditions.

#### Intervention Standardization and Fidelity

The same VR hardware, virtual environment, task, instrument settings, and guidance parameters were used throughout the study. Implementation fidelity was maintained through automated delivery of the assigned guidance condition and direct investigator supervision of each session. Performance data were automatically recorded by the VR system at the end of each session. After completing each guidance condition, participants completed the Virtual Reality System Usability Questionnaire (VRSUQ) [26].

### Outcomes

The primary outcome was the outcome error score. Secondary outcomes included the duration of operating area visibility, operating area obstruction, operator posture error, task completion time, and usability score, and were interpreted as exploratory measures of procedural performance and user experience. All quantitative performance outcomes, including outcome error score, operating area visibility, operating area obstruction, operator posture error, and task completion time, were automatically recorded and calculated by the VR simulator using predefined algorithms, thereby eliminating the need for human outcome assessment.

The outcome error score (0-15) was automatically generated using a previously validated computational assessment system [17]. The algorithm compared the final virtual access cavity with an expert-defined ideal drilling pattern of the pulp chamber walls and floor. Similarity was quantified using the *F*_1_-score and converted into a standardized clinical error scale using a supervised learning model. Previous validation demonstrated excellent agreement between automated scores and expert evaluation (Cohen κ=0.87; intraclass correlation coefficient [ICC]=0.98).

The duration of operating area visibility (seconds) was defined as the cumulative time during which the bur tip and the occlusal region of the tooth being actively prepared were visible to the operator through the dental mirror during cutting. The VR simulator continuously assessed this condition at every rendering frame using a geometric reflection-based algorithm. A vector projected from the target tooth surface toward the mirror was reflected according to the mirror orientation and compared with vectors directed from the mirror toward the left eye, right eye, and cyclops eye positions. When the minimum angular difference exceeded a predefined threshold, the target area was considered not visible through the mirror.

The duration of operator posture error was defined as the cumulative time during which the operator’s head position or orientation deviated beyond predefined thresholds from an individually calibrated reference posture. Before each session, participants established their preferred ergonomic head position and orientation, which were used as the reference for continuous posture monitoring.

The usability of the VR system was assessed using the VRSUQ [26], a validated VR-specific instrument developed to comprehensively evaluate perceived usability in immersive virtual environments. Unlike general usability scales such as the System Usability Scale (SUS) [27], which are designed for broad application across conventional systems, the VRSUQ specifically captures VR-related dimensions, including interaction, feedback, and user experience within 3D environments. The questionnaire was developed through literature review and expert input, and its construct validity was established using exploratory and confirmatory factor analyses across multiple VR systems. Responses were rated on a 5-point Likert scale and converted to a composite usability score ranging from 0 to 100, with higher scores indicating better usability.

Additional participant feedback was collected through an optional open-ended written comment following completion of the VRSUQ. The purpose of this feedback was to capture participants’ subjective impressions and suggestions regarding the different guidance modalities. Comments were reviewed by the research team and descriptively summarized to identify common observations related to usability, clarity of feedback, and perceived challenges. Given the exploratory nature and limited depth of these brief written comments, no formal qualitative coding, thematic analysis, or interrater reliability assessment was performed.

### Harms

Potential harms were defined as physical discomfort, disorientation, dizziness or motion sickness, mental burden such as tension or frustration, or other VR-related adverse effects experienced during or immediately after the simulation. Harms were assessed through direct observation by the supervising investigator, participant reporting, and prespecified items in the VRSUQ, which all participants completed after each VR session. Specifically, the VRSUQ included items assessing dizziness or motion sickness and mental burden such as tension or frustration during VR use. Participants were also instructed to report any symptoms immediately during or after each simulation session.

### Sample Size

The sample size was calculated a priori using G*Power (version 3.1.9.7) for a repeated-measures crossover design. The calculation was based on the primary outcome, outcome error score. Secondary performance outcomes were considered exploratory and were not included in the a priori sample size estimation. Assuming a medium effect size (Cohen *f*=0.25), an α level of .05, a power of 90%, and an assumed correlation among repeated measures of 0.60, a minimum sample size of 25 participants was required. This approach was consistent with a previous VR-based dental education study involving similar learner populations and performance-based outcomes, although that study did not specifically evaluate mirror guidance [28]. To account for potential participant withdrawal or incomplete datasets, 30 participants were recruited. No interim analyses were planned or conducted, and no stopping guidelines were specified.

### Randomization

#### Sequence Generation

Participants were randomly assigned to 1 of 4 intervention sequences according to a balanced 4 × 4 Latin square crossover design. The random allocation sequence was generated by an independent statistician who was not involved in the study design or participant recruitment. Restricted randomization using the balanced Latin square design ensured that each participant completed all 4 intervention conditions (verbal guidance, visual guidance, haptic guidance, and no guidance) in a predefined sequence, with each condition appearing once in each study period across the 4 predefined sequences to control for potential order effects.

#### Allocation Concealment Mechanism

Allocation concealment was achieved using sequentially numbered, sealed, opaque envelopes containing the randomized intervention sequences. The envelopes were opened only after participant enrollment to maintain concealment of sequence allocation.

#### Implementation

Participants were enrolled by the research team, who did not have access to the random allocation sequence before enrollment. After enrollment, intervention sequences were assigned by the research team according to the sequentially numbered, sealed, opaque envelopes. The allocation sequence remained concealed until the corresponding envelope was opened after enrollment.

#### Blinding

Participant blinding was not feasible because the 4 VR mirror guidance conditions (verbal, visual, haptic, and no guidance) were inherently distinguishable during the intervention. Investigators supervising the VR sessions were also aware of the assigned guidance conditions. Participants were not informed that any specific guidance modality was considered the intervention of primary interest or that the no-guidance condition served as the comparator. Performance outcomes were automatically recorded by the VR simulator, reducing the potential for assessor-related bias. Statistical analyses were performed by an independent statistician who was not involved in the study design and was blinded to the intervention labels.

### Statistical Methods

Statistical analyses were performed using IBM SPSS Statistics (IBM Corp). A linear mixed-effects model was used to evaluate the effects of VR guidance condition (verbal, visual, haptic, and no guidance) on performance and usability outcomes within the 4 × 4 Latin square crossover design. The model included fixed effects for treatment (guidance condition), period, and sequence, with participants treated as a random effect to account for repeated measurements within individuals. A compound symmetry covariance structure was applied to model the correlation among repeated observations from the same participant.

Because the study was designed with a single predefined primary outcome, the significance threshold was set at α=.05 for the primary analysis. Secondary outcomes were considered exploratory, and no formal adjustment for multiplicity was applied across secondary outcomes. Model assumptions were assessed through residual diagnostics, including visual inspection of residual plots and evaluation of residual normality using the Shapiro-Wilk test. Treatment effects are reported as *F* statistics, *P* values, and partial eta-squared (ηp^2^) effect sizes calculated from the corresponding *F* statistics and degrees of freedom. Estimated marginal mean differences (MDs) with 95% CIs and ηp^2^ effect sizes were reported, with Bonferroni-adjusted pairwise comparisons used to account for multiple comparisons. A *P* value of <.05 was considered statistically significant.

## Results

### Participant Flow

Thirty fourth-year preclinical dental students were assessed for eligibility. No students met the exclusion criterion of prior experience with a dental virtual reality simulator. All 30 eligible students provided written informed consent and were enrolled in the study (Figure 4). Participants were randomly assigned to 1 of 4 intervention sequences according to a balanced 4 × 4 Latin square design and completed all 4 experimental conditions (verbal guidance, visual guidance, haptic guidance, and no guidance) across 4 sessions separated by 48-hour washout intervals. All randomized participants completed the study, with no protocol deviations, intervention discontinuations, missing outcome data, or losses to follow-up. All 30 randomized participants were included in the final analysis.

**Figure 4:**
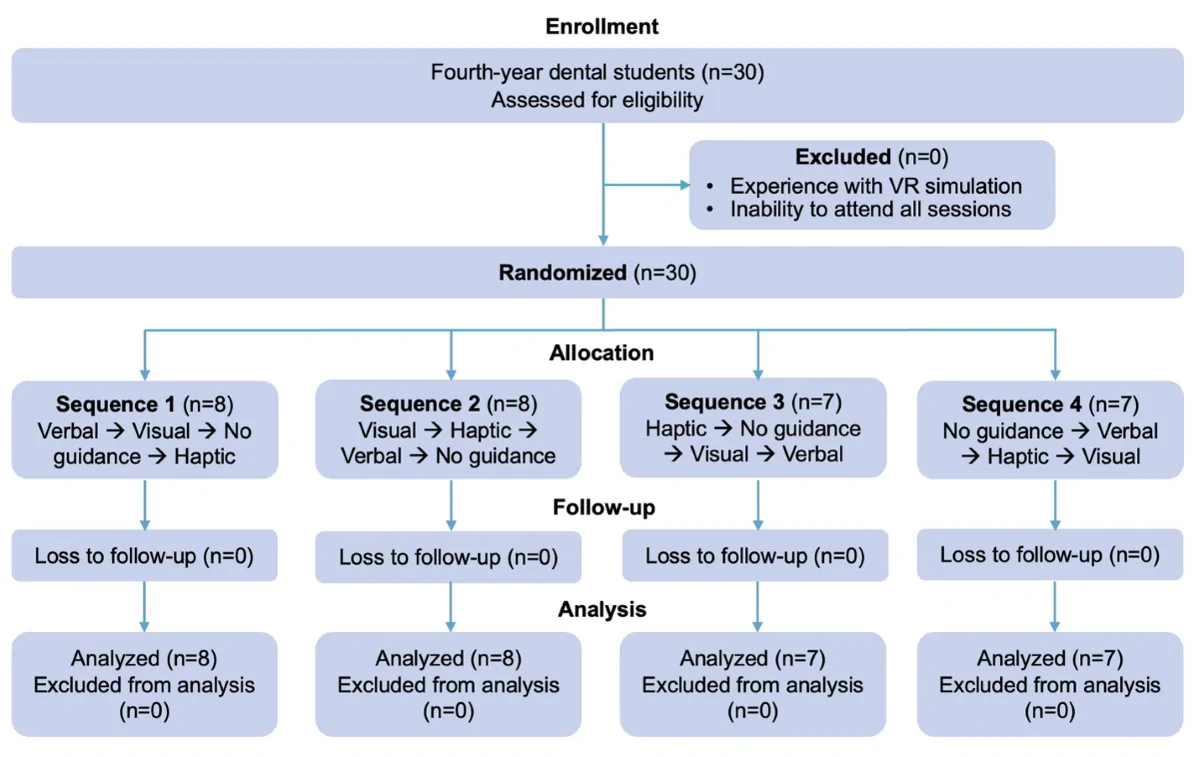
CONSORT (Consolidated Standards of Reporting Trials) flow diagram showing participant progression through the randomized controlled crossover study. Thirty fourth-year dental students were assessed for eligibility and enrolled. Participants were randomized to 1 of 4 sequences in a 4 × 4 Latin square crossover design and completed all 4 virtual reality (VR) mirror guidance conditions (verbal, visual, haptic, and no guidance). No participants withdrew, and data from all 30 participants were included in the final analysis.

### Recruitment

Participants were recruited through email invitations and public announcements from July 18 to 23, 2026. Participation was voluntary, and students were free to decline participation without any academic consequences. Outcome and harms assessments were conducted during the experimental sessions from July 25, 2026, to September 4, 2026, with harms assessed during and immediately after each session. The trial ended as planned after all enrolled participants had completed the prespecified study procedures; it was not stopped early.

### Intervention and Comparator Delivery

All guidance conditions and the no-guidance comparator were delivered as intended by the VR system, with no deviations from the assigned conditions. The supervising investigator provided standardized orientation and technical support but did not provide additional instructional guidance or performance feedback during the experimental tasks. No concomitant training or other educational interventions related to indirect vision or VR mirror guidance were provided during the trial.

### Baseline Data

Thirty participants completed the study (female: n=21, 70%; male: n=9, 30%). The mean age was 22.7 (SD 1.62; range 21-26) years. Twenty-five (83.33%) participants reported myopia and performed the VR sessions using their habitual visual correction (eyeglasses or contact lenses) while wearing the head-mounted display, whereas 5 (16.67%) participants reported normal vision. All participants were right-handed. All participants completed the 4 guidance conditions according to the assigned Latin square sequence (Table 1).

**Table 1:** Baseline characteristics of fourth-year preclinical dental students enrolled in a randomized controlled crossover trial evaluating virtual reality–based mirror guidance for indirect vision training during minimally invasive endodontic access cavity preparation (n=30).

Characteristic	Value
Age (years), mean (SD)	22.7 (1.62)
Female, n (%)	21 (70)
Male, n (%)	9 (30)
Myopia, n (%)	25 (83.33)
Normal vision, n (%)	5 (16.67)
Right-handed, n (%)	30 (100)

### Numbers Analyzed, Outcomes, and Estimation

All 30 randomized participants were included in the analysis of each outcome, with no missing outcome data. Linear mixed-effects analysis demonstrated a significant effect of guidance modality on the primary outcome, outcome error score (*F*_3,84_=20.34; *P*<.001; ηp^2^=0.421). Mean outcome error scores were 2.57 (SD 0.52) for verbal guidance, 2.39 (SD 0.31) for visual guidance, 2.44 (SD 0.38) for haptic guidance, and 3.07 (SD 0.30) for no guidance. Bonferroni-adjusted pairwise comparisons showed significantly lower outcome error scores for verbal (MD −0.50, 95% CI −0.761 to −0.235; adjusted *P*<.001), visual (MD −0.68, 95% CI −0.944 to −0.417; adjusted *P*<.001), and haptic guidance (MD −0.63, 95% CI −0.892 to −0.365; adjusted *P*<.001) compared with no guidance.

Guidance modality also had a significant effect on the duration of operating area visibility (*F*_3,84_=12.31; *P*<.001; ηp^2^=0.305). Mean visibility durations were 131.12 (SD 54.29) seconds for verbal guidance, 152.38 (SD 33.68) seconds for visual guidance, 124.79 (SD 46.35) seconds for haptic guidance, and 93.36 (SD 33.98) seconds for no guidance. Bonferroni-adjusted comparisons showed significantly longer durations of operating area visibility with verbal guidance (MD 37.79, 95% CI 11.15-64.43 seconds; adjusted *P*=.001), visual guidance (MD 59.15, 95% CI 32.51-85.79 seconds; adjusted *P*<.001), and haptic guidance (MD 31.52, 95% CI 4.90-58.15 seconds; adjusted *P*=.01) compared with no guidance.

No statistically significant treatment effects were detected for operating area obstruction (*F*_3,84_=1.94; *P*=.13; ηp^2^=0.065), operator posture error (*F*_3,84_=0.93; *P*=.43; ηp^2^=0.032), task completion time (*F*_3,84_=1.73; *P*=.17; ηp^2^=0.058), or usability score (*F*_3,84_=0.72; *P*=.54; ηp^2^=0.025). Descriptively, operating area obstruction was lowest with verbal guidance (54.92, SD 45.39 seconds) and highest with no guidance (72.96, SD 44.02 seconds). Haptic guidance had the lowest mean duration of operator posture error (1.54, SD 1.04 seconds), while task completion time was shortest with haptic guidance (212.47, SD 56.43 seconds). Usability scores were high and similar across all conditions, ranging from 83.48 (SD 8.99) with verbal guidance to 85.37 (SD 8.23) with visual guidance.

No significant period effects were detected for outcome error score (*P*=.62), operating area visibility (*P*=.78), operating area obstruction (*P*=.38), operator posture error (*P*=.64), task completion time (*P*=.79), or usability score (*P*=.19). Similarly, no significant sequence effects were identified for any outcome (all *P*>.05), indicating no evidence of systematic period or sequence effects in the crossover design (Table 2).

Figure 5 illustrates the distribution of performance and usability outcomes across the 4 guidance conditions.

**Table 2:** Performance and usability outcomes across 4 virtual reality mirror guidance conditions (verbal, visual, haptic, and no guidance) in a randomized controlled crossover trial of indirect vision training for minimally invasive endodontic access cavity preparation among fourth-year preclinical dental students (n=30). F test values correspond to the fixed effect of guidance modality from the linear mixed-effects models accounting for the 4 × 4 Latin square crossover design.

Outcome	Verbal, mean (SD)	Visual, mean (SD)	Haptic, mean (SD)	No guidance, mean (SD)	*F* test (*df*)	Treatment *P* value	Period *P* value	Sequence *P* value	Effect size, ηp2
Outcome error score (0-15)	2.57 (0.52)	2.39 (0.31)	2.44 (0.38)	3.07 (0.30)	20.34 (3, 84)	<.001	.62	.64	0.421
Duration of operating area visibility (seconds)	131.12 (54.29)	152.38 (33.68)	124.79 (46.35)	93.36 (33.98)	12.31 (3, 84)	<.001	.78	.22	0.305
Duration of operating area obstruction (seconds)	54.92 (45.39)	59.07 (39.84)	57.46 (37.21)	72.96 (44.02)	1.94 (3, 84)	.13	.38	.80	0.065
Duration of operator posture error (seconds)	1.75 (1.19)	1.74 (1.03)	1.54 (1.04)	2.00 (1.01)	0.93 (3, 84)	.43	.64	.93	0.032
Task completion time (seconds)	232.68 (61.55)	219.06 (65.15)	212.47 (56.43)	232.37 (48.66)	1.73 (3, 84)	.17	.79	.40	0.058
Usability score (0-100)	83.48 (8.99)	85.37 (8.23)	83.74 (8.30)	84.11 (8.78)	0.72 (3, 84)	.54	.19	.99	0.025

**Figure 5:**
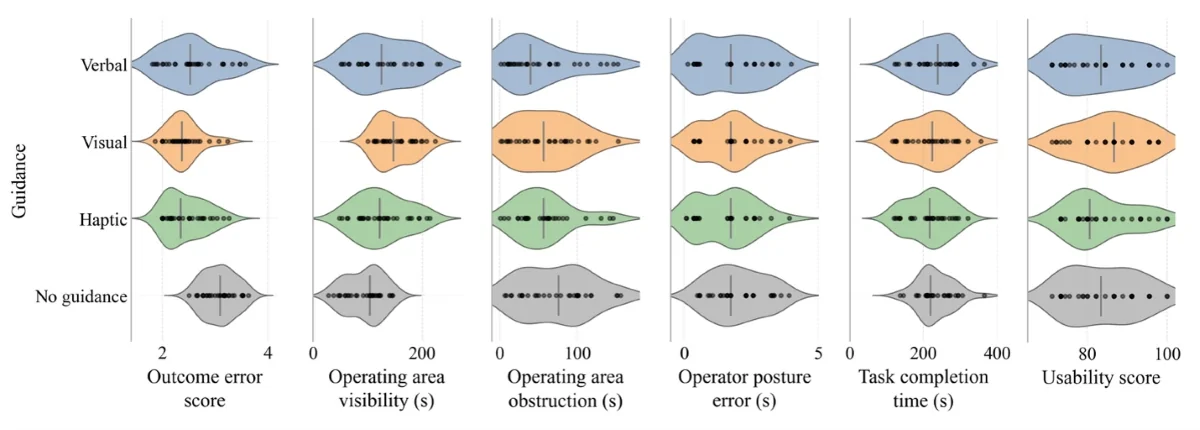
Distribution of performance and usability outcomes across 4 virtual reality mirror guidance conditions (verbal guidance, visual guidance, haptic guidance, and no guidance) in a randomized controlled crossover trial of indirect vision training for minimally invasive endodontic access cavity preparation among fourth-year preclinical dental students. Violin plots display the distribution density of individual participant values for outcome error score, operating area visibility, operating area obstruction, operator posture error, task completion time, and usability score.

### Harms

No important harms or unintended effects were detected during any of the 4 VR mirror guidance conditions. No participants reported physical discomfort, disorientation, motion sickness, or other adverse reactions related to the VR simulation. No unintended positive or negative effects that influenced study participation or outcomes were identified.

### Ancillary analyses

The VRSUQ demonstrated good internal consistency in the present study (Cronbach α=0.854), supporting the reliability of the usability measurements obtained from the participants. Table 3 presents the mean VRSUQ scores across the 4 guidance conditions. Overall, participants reported positive usability perceptions for all conditions, with mean item scores generally above 3.75 on the 5-point scale.

**Table 3:** VRSUQ_a_ scores across 4 VR_b_ mirror guidance conditions (verbal, visual, haptic, and no guidance) in a randomized controlled crossover trial of indirect vision training for minimally invasive endodontic access cavity preparation among fourth-year preclinical dental students (n=30). Responses were rated on a 5-point Likert scale (1=strongly disagree; 5=strongly agree).

Item	Description	Verbal, mean (SD)	Visual, mean (SD)	Haptic, mean (SD)	No guidance, mean (SD)
1	System responded well to user manipulations without delays	4.40 (0.61)	4.40 (0.55)	4.35 (0.59)	4.40 (0.62)
2	VR system provided clear feedback on manipulations	4.45 (0.59)	4.60 (0.54)	4.40 (0.61)	4.45 (0.64)
3^c^	I kept making errors while using the VR system	4.50 (0.57)	4.70 (0.49)	4.45 (0.56)	4.50 (0.60)
4	Information presented in the virtual environment was easy to understand	3.85 (0.58)	3.85 (0.64)	3.95 (0.51)	4.00 (0.69)
5	The system was user-friendly and easy to learn	4.00 (0.68)	3.90 (0.58)	4.00 (0.63)	4.10 (0.72)
6	It was easy to correct errors during VR use	4.45 (0.56)	4.60 (0.48)	4.50 (0.55)	4.40 (0.61)
7	I enjoyed the virtual reality experience	4.40 (0.59)	4.60 (0.51)	4.60 (0.58)	4.40 (0.63)
8^c^	I experienced dizziness or motion sickness during VR use	4.50 (0.55)	4.60 (0.47)	4.50 (0.54)	4.60 (0.59)
9^c^	I experienced mental burden such as tension or frustration during VR use	4.50 (0.57)	4.50 (0.50)	4.45 (0.57)	4.50 (0.60)
Total	Overall usability score (1-100)	83.48 (8.99)	85.37 (8.23)	83.74 (8.30)	84.11 (8.78)

^a^VRSUQ: Virtual Reality System Usability Questionnaire.

^b^VR: virtual reality.

^c^Items were reverse-coded according to the questionnaire developer [27] so that higher scores consistently indicated more positive usability. The composite usability score (0-100) was calculated as: (mean item score − 1)/4 × 100.

Participants were invited to provide additional comments following completion of the usability questionnaire. These comments were reviewed and summarized descriptively to identify recurring perceptions regarding each guidance modality. Several common observations were identified regarding the perceived advantages, challenges, and areas for improvement of each feedback strategy. Verbal guidance was generally perceived as supportive and reassuring, with participants noting that it resembled continuous instructor supervision. The auditory reminders that the bur was not visible were described as helpful for maintaining awareness of the operative field, although some participants reported uncertainty regarding the required level of visibility. For visual guidance, some participants reported that the combination of multiple visual elements (eg, ghost mirror, reflection line, and cone visualization) was complex and occasionally confusing. For haptic guidance, some participants noted that although corrective forces were perceived, the direction of the required mirror adjustment was not always clear. Overall, the qualitative feedback aligns with the usability findings and highlights areas for refinement, particularly for visual and haptic guidance modalities.

## Discussion

### Principal Findings

This randomized controlled crossover trial evaluated the effectiveness and usability of different VR-based mirror guidance modalities, including verbal, visual, and haptic guidance, for improving indirect vision during minimally invasive endodontic access cavity preparation. All 3 guidance modalities significantly reduced outcome error scores compared with no guidance. Verbal, visual, and haptic guidance also significantly increased operating area visibility. In contrast, operating area obstruction, operator posture error, task completion time, and usability did not differ significantly among conditions. Together, these findings suggest that real-time mirror guidance can improve key aspects of indirect vision performance without compromising task efficiency or perceived usability, supporting its integration into VR-based dental training [29,30].

### Comparison With Prior Work

Recent work by Samuel et al [23] on visuo-haptic guidance for dental nerve block training provides an important reference point for the design of multimodal feedback in immersive dental simulation. Their system integrates stepwise visual guidance with haptic feedback to support procedural learning, using a gated progression approach in which learners must align a virtual syringe with predefined spatial targets before advancing through the task. In contrast, the present study addresses a different but equally critical challenge in dental education—real-time mirror positioning for indirect vision, which is inherently continuous, dynamic, and less amenable to discrete stepwise guidance. Rather than guiding learners through predefined procedural stages, our visual guidance system supports ongoing spatial alignment between the mirror, operator viewpoint, and operative field. This distinction is important: while prior systems emphasize procedural correctness, our approach targets perceptual-motor coordination under indirect visualization conditions [17].

Differences in task structure may explain the contrasting usability findings. The gated, sequential guidance of Samuel et al [23] may reduce extraneous cognitive load by limiting concurrent visual information, whereas multiple visual elements in the present study may have increased processing demands during mirror interpretation and fine motor control [12,19]. Consistent with Cognitive Load Theory [19], participant feedback identified visual complexity as a source of confusion despite improved operating area visibility; however, cognitive load was not directly measured. Thus, stepwise guidance may suit sequential procedures [23], whereas simplified or adaptive guidance may be more appropriate for continuous perceptual-motor tasks such as indirect vision [12,19]. Future systems could dynamically adjust visual cues according to task phase or learner proficiency.

For haptic guidance, some participants noted that although corrective forces were perceived, the direction of the required mirror adjustment was not always clear. This limitation may be attributable to the characteristics of the haptic interface used in this study. The dental virtual handpiece and mirror were controlled using a Geomagic Touch device, which provides 6 degrees of freedom positional input but only 3 degrees of freedom force output. While users can manipulate the mirror in both translation and rotation, the system can deliver force feedback only in translational directions and cannot provide rotational torque cues. From a motor learning perspective, ambiguous or incomplete feedback may impair accurate error attribution and hinder efficient motor adaptation [18]. The use of a fully actuated 6 degrees of freedom haptic device capable of delivering both force and torque feedback may therefore improve spatial specificity and instructional effectiveness [31].

Lee and Choi [24] demonstrated that haptic disturbance can enhance motor learning by promoting active error correction, whereas the present system primarily used assistive corrective forces. Controlled perturbation or progressive fading of guidance may therefore promote active learning and skill retention [18,24]. Participants also reported surprise when haptic correction was activated, suggesting that abrupt feedback may disrupt ongoing motor execution. Coordinated multimodal feedback may facilitate skill acquisition when sensory channels provide complementary information [18,20,23,24]. Although these mechanisms were not directly evaluated, anticipatory verbal cues before haptic activation may facilitate sensorimotor integration and, consistent with Multimedia Learning Theory [20], support cognitive processing and motor learning.

The challenges associated with indirect vision are not unique to dentistry. Similar visuomotor demands are encountered in laparoscopic and microsurgical procedures, where surgeons must interpret indirect visual information and translate it into precise instrument movements [32-34]. Recent advances in these fields have highlighted the importance of augmented visual feedback to improve spatial orientation and facilitate skill acquisition. For example, Feng et al [32] demonstrated that a virtual pointer for gaze guidance during laparoscopic surgery improved users’ ability to maintain visual attention on relevant anatomical targets and enhanced task efficiency. Similarly, Gao et al [33] developed an intraoperative laparoscopic photoacoustic image guidance system integrated with the da Vinci surgical platform to provide additional real-time visual information that supports more accurate localization of anatomical structures. In microsurgical ophthalmic training, Akimoto et al [34] showed that recording and sharing the direct microscopic view of experienced surgeons provided trainees with improved visual references and enhanced understanding of microsurgical maneuvers. Although these technologies differ from the present VR mirror guidance system, they collectively support the concept that supplementary visual information can reduce the challenges associated with indirect visualization and facilitate the development of complex visuomotor skills [32-34]. The present findings extend this concept to dental education, supporting real-time VR guidance for indirect vision and the adaptation of guidance strategies from image-dependent surgical disciplines to psychomotor dental training.

### Strengths and Limitations

The significant improvements in both outcome error scores and operating area visibility suggest that VR-based mirror guidance can enhance procedural accuracy and maintenance of the operative field, while the absence of significant differences in other performance measures indicates that these benefits did not compromise task efficiency or usability. Participant feedback provided complementary insights into the usability, interpretability, and perceived demands of each guidance modality. By building upon an established and validated VR simulation platform and introducing targeted feedback enhancements, the present study isolates the pedagogical contribution of multimodal mirror guidance, minimizing potential confounding effects related to system novelty. The integration of quantitative performance data with qualitative user feedback therefore strengthens both the ecological validity and the practical relevance of the findings for the design of VR-based dental training systems.

Several limitations should be considered. First, participants were fourth-year dental students from a single institution, limiting generalizability. Although adequately powered for the primary outcome, confidence intervals indicate some uncertainty in effect magnitude, and smaller differences in exploratory secondary outcomes may have remained undetected. As no multiplicity adjustment was applied across the exploratory secondary outcomes, nonsignificant findings should not be interpreted as evidence of equivalence. Replication in larger, independent, and more diverse learner populations is warranted. Second, corrected visual acuity and refractive error were not formally assessed, precluding evaluation of their potential influence on VR performance. Third, although the Latin square design balanced condition order and no period or sequence effects were detected, repeated use of the same virtual tooth may have introduced anatomical familiarity or task-specific learning. Fourth, the standardized simulated environment cannot fully reproduce clinical complexity; therefore, the findings reflect short-term training performance rather than clinical effectiveness or direct translation to patient care [8,29,30]. In addition, the haptic system provided 3 degrees of freedom force feedback without rotational torque, potentially limiting haptic guidance for mirror orientation [31]. Further clinical validation is warranted. Fifth, each guidance modality incorporated multiple feedback components, preventing determination of the contribution of individual elements. Sixth, although the VRSUQ showed good internal consistency, further validation in dental VR contexts is warranted [26]. Finally, participant comments were obtained through brief open-ended responses rather than structured qualitative interviews and should therefore be considered exploratory. As the study involved preclinical students in a standardized simulated environment, the findings reflect short-term training performance rather than clinical effectiveness, and further clinical validation is needed.

### Future Directions

Future research should include larger, independent, and more diverse cohorts and longitudinal designs to evaluate skill retention, transfer, and clinical performance. Factorial or component-ablation studies could clarify the contribution of individual feedback elements. Fully actuated 6 degrees of freedom haptic devices providing force and torque feedback may improve haptic guidance, while simplified, adaptive, or user-specific visual cues may balance performance benefits with usability. Hybrid strategies combining modalities sequentially or adaptively, including progressive fading of assistance, also warrant investigation.

### Conclusions

VR-based mirror guidance improved the outcome error score and increased operating area visibility during minimally invasive endodontic access cavity preparation. Verbal, visual, and haptic guidance resulted in fewer outcome errors and greater operating area visibility than no guidance, while no significant differences were detected in other performance measures or usability. These findings demonstrate the potential of diverse feedback modalities to provide effective guidance for indirect vision and inform future development of adaptive VR training systems using one or more modalities to suit learner needs.

## Appendix

Multimedia Appendix 1 CONSORT 2025 checklist.

Multimedia Appendix 2 CONSORT-EHEALTH (V 1.6.1) checklist.

Multimedia Appendix 3 Virtual reality–based mirror guidance system source code.

Multimedia Appendix 4 Virtual reality–based mirror guidance system screenshots.

## Abbreviations

CONSORT: Consolidated Standards of Reporting Trials
CONSORT-EHEALTH: Consolidated Standards of Reporting Trials of Electronic and Mobile Health
ICC: intraclass correlation coefficient
SUS: System Usability Scale
UE: Unreal Engine
VR: virtual reality
VRSUQ: Virtual Reality System Usability Questionnaire

## Data Availability

The source code for the VR-based mirror guidance techniques and a demonstration of the system are available in Multimedia Appendices 3 and 4 and online [35].
