# Supplementary material for: Virtual Reality–Based Mirror Guidance for Indirect Vision Training in Minimally Invasive Endodontic Access Cavity Preparation Among Dental Students: Randomized Controlled Crossover Trial
Source: JMIR Serious Games. 2026 Oct 1;14:e100884. doi: 10.2196/100884 (PMC13673179; doi:10.2196/100884)
Supplement: Supplementary file 3 [file games_v14i1e100884_app3.docx]

**VR-based mirror guidance system source code**

// Methods Explanation

- Tick(float DeltaTime):
  - Loop function.
  - Call every frame
  - Parameter:
    - **DeltaTime:** The time between the last frame and the current frame in second.
- FindSignedAngleBetween2DVectors(FVector2D a, FVector2D b)
  - Find a signed angle between two given vectors ***a*** and ***b***
- GetReflectionVector(FVector sorceLocation, FVector reflectionPoint, FVector reflectionPointNormal)
  - Find the reflection vector from the source on the given surface
  - Parameters
    - **sorceLocation**: The source location of the reflection
    - **reflectionPoint**: The location where the reflection occurs
    - **reflectionPointNormal**: The normal vector on the reflection location
- ChangeCurrentGuidance():
  - Switching the current guidance method
- FindRouteAroundSphere(const FVector& PointA, const FVector& PointD, const FVector& SphereCenter, float SphereRadius)
  - Find the tangent line from the **PointA** on the given **Sphere**, when the occlusion between **PointA** and **PointD** occurs
  - Parameters
    - **PointA**: The source location
    - **PointD**: The target location
    - **SphereCenter**: The center of the sphere
    - **SphereRadius**: The radius of the sphere
- GetPerpendicularInPlane(const FVector& Primary, const FVector& Reference)
  - Find the perpendicular vector from the **Primary** vector projecting toward the **Reference** vector
- IsInsideACone(FVector origin, FVector direction, float radius1, float radius2, float coneAngle, FVector point)
  - Check if the given **point** is inside the optimal area or not
  - Parameters:
    - **origin**: The origin of the cone
    - **direction**: The direction of the cone
    - **radius1**: The inner radius
    - **radius2**: The outer radius
    - **coneAngle**: The cone angle
    - **point**: The target location

// --------------------------------------------------------------------

#define CONE_ANGLE_SUBTRACTOR 10.0f
#define ANGLE_THRESHOLD 20.0f // Angle threshold for applying guidance #define MAX_SOUND_PLAY_TIME 2
#define SOUND_DELAY_TIME 2.0f
#define HAPTIC_DISTANCE_THRESHOLD 0.5f
#define HAPTIC_CONE_STIFFNESS 0.5f
#define HAPTIC_MOVEMENT_TYPE1_STIFFNESS 7.0f
#define HAPTIC_MOVEMENT_TYPE3_STIFFNESS 0.7f

// Called every frame

void AGuidanceActor::Tick(float DeltaTime)

{

Super::Tick(DeltaTime);

```
// Begin -- Adjust Position
// Posture Checking
if (postureCheckerActor && !(postureCheckerActor->GetActorLocation().Equals(FVector(0.0f), 0.01f)))
{
    float posturePositionalChecker = FVector::Dist(head->GetComponentLocation(), postureCheckerActor->GetActorLocation());
    float postureRotationalChecker = UKismetMathLibrary::DegAcos(FVector::DotProduct(head->GetForwardVector(), postureCheckerActor->GetActorForwardVector()));
    if (posturePositionalChecker > 9.0f || postureRotationalChecker > 25.0f)
    {
        durationIncorrectPostureCounter += DeltaTime;

        if (!isSoundPlaying)
        {
            isSoundPlaying = true;
            USoundBase* soundToPlay = (reminderCounters[1]++ > MAX_SOUND_PLAY_TIME) ? reminderSounds[2] : reminderSounds[3];

            UGameplayStatics::PlaySound2D(GetWorld(), soundToPlay);
            GetWorldTimerManager().SetTimer(timeHandler[0], resetSoundInstructionDelegate, soundToPlay->GetDuration() + SOUND_DELAY_TIME, false);
        }
    }
    else if (durationIncorrectPostureCounter > 0.0f)
    {
        individualIncorrectPosture.Add(durationIncorrectPostureCounter);
        durationIncorrectPostureCounter = 0.0f;
    }

    if (triggerActor == nullptr)
    {
        // Evaluation - 1
        timeToFinishTheSimulation += DeltaTime;


        // Provide guidance only when the tool tip is closed to the tooth
        if (FVector::Dist(toolTip->GetComponentLocation(), activeTooth->GetActorLocation()) < 1.0f)
        {
            // Play sonund when the tool tip is not visible in the mirror
            FHitResult traceResult;
            TArray<AActor*> ignoredActor;
            ignoredActor.Add(activeTooth);

            FVector A = toothTip->GetComponentLocation();
            FVector B = toolBlocker->GetComponentLocation();
            float C = toolBlocker->GetScaledSphereRadius();
            FVector D = mirror->MirrorReflectionMesh->GetComponentLocation();
            FVector D_prime = FindRouteAroundSphere(A, D, B, C);
            bool seeToolTip = D_prime.Equals(D);

            // Rotate Ghost tool
            FVector r = GetReflectionVector(toothTip->GetComponentLocation(), reflector->GetComponentLocation(), reflector->GetUpVector());

            FVector targetVector = head->GetComponentLocation() - reflector->GetComponentLocation();
            targetVector.Normalize();

            float angle = UKismetMathLibrary::DegAcos(FVector::DotProduct(targetVector, r));

            if (!inRange && angle < 2.5f) inRange = true;
            else if (inRange && angle > 5.0f) inRange = false;

            if (!inRange)
            {
                FVector2D targetVector2D[3] =
                {
                    FVector2D(targetVector.X, targetVector.Y),
                    FVector2D(targetVector.X, targetVector.Z),
                    FVector2D(targetVector.Y, targetVector.Z)
                };

                FVector2D reflectionVector2D[3] =
                {
                    FVector2D(r.X, r.Y),
                    FVector2D(r.X, r.Z),
                    FVector2D(r.Y, r.Z)
                };

                float angle2D[3] =
                {
                    FindSignedAngleBetween2DVectors(targetVector2D[0], reflectionVector2D[0]),        // Roll
                    FindSignedAngleBetween2DVectors(targetVector2D[1], reflectionVector2D[1]),        // Pinch
                    FindSignedAngleBetween2DVectors(targetVector2D[2], reflectionVector2D[2])        // Yall
                };

                FRotator newRotation = GetActorRotation() + FRotator(angle2D[1] * DeltaTime * 15.0f, angle2D[0] * DeltaTime * 15.0f, 0.0f);

                angle = UKismetMathLibrary::DegAcos(FVector::DotProduct(UKismetMathLibrary::GetForwardVector(initialRotation), UKismetMathLibrary::GetForwardVector(newRotation)));
                if (angle < 100.0f)
                {
                    this->AddActorLocalRotation(FRotator(angle2D[1] * DeltaTime * 15.0f, angle2D[0] * DeltaTime * 15.0f, 0.0f));
                }
                else
                {
                    this->SetActorRotation(initialRotation);
                }

            }

            // Calculate Angle for the Mirror Tool
            float mirrorToothDistance = FVector::Dist(toothTip->GetComponentLocation(), mirror->MirrorReflectionMesh->GetComponentLocation());
            float currentAngleThreshold;
            if (mirrorToothDistance >= 2.4f)
            {
                currentAngleThreshold = ANGLE_THRESHOLD - (angleSubtractorMultiplier * mirrorToothDistance);
            }
            else
            {
                currentAngleThreshold = ANGLE_THRESHOLD + (angleSubtractorMultiplier * mirrorToothDistance);
            }

            r = GetReflectionVector(toothTip->GetComponentLocation(), mirror->MirrorReflectionMesh->GetComponentLocation(), mirror->MirrorReflectionMesh->GetUpVector());
            float t = 50.0f;
            FVector p_t = mirror->MirrorReflectionMesh->GetComponentLocation() + (t * r);

            FVector targetVectors[3] =
            {
                head->GetComponentLocation() - mirror->MirrorReflectionMesh->GetComponentLocation(),
                leftEye->GetComponentLocation() - mirror->MirrorReflectionMesh->GetComponentLocation(),
                rightEye->GetComponentLocation() - mirror->MirrorReflectionMesh->GetComponentLocation()
            };
            angle = INFINITY;
            for (FVector vec : targetVectors)
            {
                vec.Normalize();
                float tmpAngle = UKismetMathLibrary::DegAcos(FVector::DotProduct(vec, r));
                if (tmpAngle < angle) angle = tmpAngle;
            }

            textRenderActor->GetTextRender()->SetText(FText::FromString(FString::SanitizeFloat(angle)));
            textRenderActor2->GetTextRender()->SetText(FText::FromString(FString::SanitizeFloat(mirrorToothDistance)));
            textRenderActor3->GetTextRender()->SetText(FText::FromString(FString::SanitizeFloat(currentAngleThreshold)));

            // Evaluation - 2
            if (durationNoReflectCounter > 0.0f && angle < currentAngleThreshold)
            {
                individualDurationNoReflect.Add(durationNoReflectCounter);
                durationNoReflectCounter = 0.0f;
            }
            else if (angle > currentAngleThreshold) // tooth is not in the mirror
            {
                durationNoReflectCounter += DeltaTime;
            }

            if (duratioBlockedCounter > 0.0f && seeToolTip)
            {
                individualDurationBlocked.Add(duratioBlockedCounter);
                duratioBlockedCounter = 0.0f;
            }
            else if (!seeToolTip)
            {
                duratioBlockedCounter += DeltaTime;
            }

            if (angle < currentAngleThreshold && seeToolTip)
            {
                durationToothVisible += DeltaTime;
            }

            // use if statement since switch case does not support local variable declaration
            if (currentGuidanceMethod == EGuidanceMethod::CombineGuidance || currentGuidanceMethod == EGuidanceMethod::Visual)
            {
                if (IsInsideACone(coneStartActor->GetActorLocation(), coneStartActor->GetActorForwardVector(), 2.0f, 20.0f, hapticConeAngle, mirror->MirrorReflectionMesh->GetComponentLocation()))
                {
                    if (coneMesh->IsVisible())
                    {
                        // Hide cone
                        coneMesh->SetVisibility(false);

                        // show ghost actor and line
                        this->SetActorHiddenInGame(false);
                        lineGuidanceActors[0]->SetActorHiddenInGame(true);
                        lineDynMAT->SetVectorParameterValue("Color", FLinearColor::Blue);
                    }

                    FVector targetVisibilityLocation = toothTip->GetComponentLocation();
                    FVector blockerLocation = toolBlocker->GetComponentLocation();
                    float blockerRadius = toolBlocker->GetScaledSphereRadius() * 1.1f;
                    FVector mirrorLocation = mirror->MirrorReflectionMesh->GetComponentLocation();
                    FVector newMirrorLocation = FindRouteAroundSphere(targetVisibilityLocation, mirrorLocation, blockerLocation, blockerRadius);


                    float opacity = 0.0f;
                    if (!seeToolTip)
                    {
                        if (!lineGuidanceActors[1]->IsHidden())
                        {
                            lineGuidanceActors[1]->SetActorHiddenInGame(true);
                        }
                        opacity = 0.1f;

                        FVector positionDifference = newMirrorLocation - reflector->GetComponentLocation();
                        this->AddActorWorldOffset(positionDifference);
                    }
                    else
                    {
                        // Move the ghost location to the actual mirror location
                        FVector positionDifference = mirror->MirrorReflectionMesh->GetComponentLocation() - reflector->GetComponentLocation();
                        this->AddActorWorldOffset(positionDifference);

                        if (lineGuidanceActors[1]->IsHidden())
                        {
                            lineGuidanceActors[1]->SetActorHiddenInGame(false);
                        }

                        opacity = FMath::Clamp((0.01f * angle) - (currentAngleThreshold) / 100.0f, 0.0f, 0.1f);
                    }

                    // Linear interpotaiton to detemine opticity of visual guidance
                    mirrorDynMAT->SetScalarParameterValue(TEXT("Opacity"), opacity);
                    lineDynMAT->SetScalarParameterValue(TEXT("Opacity"), opacity * 10.0f);

                    // Move Line
                    FRotator toothToMirrorRotation = UKismetMathLibrary::FindLookAtRotation(lineGuidanceActors[0]->GetActorLocation(), mirror->MirrorReflectionMesh->GetComponentLocation());
                    FRotator mirrorReflectionRotation = UKismetMathLibrary::FindLookAtRotation(lineGuidanceActors[1]->GetActorLocation(), p_t);

                    lineGuidanceActors[0]->SetActorRotation(toothToMirrorRotation);
                    lineGuidanceActors[1]->SetActorRotation(mirrorReflectionRotation);

                    lineGuidanceActors[0]->SetActorScale3D(FVector(FVector::Distance(lineGuidanceActors[0]->GetActorLocation(), mirror->MirrorReflectionMesh->GetComponentLocation()), 1.0f, 1.0f));
                }
                else
                {
                    if (!coneMesh->IsVisible())
                    {
                        // Show cone
                        coneMesh->SetVisibility(true);

                        // Hide ghost actor and line
                        this->SetActorHiddenInGame(true);
                        lineGuidanceActors[0]->SetActorHiddenInGame(true);

                        lineDynMAT->SetVectorParameterValue("Color", FLinearColor::Green);
                        lineDynMAT->SetScalarParameterValue(TEXT("Opacity"), 0.5f);
                    }

                    float coneTargetDistance = (hapticSmallRadius + 1.0);
                    targetLocation = coneStartActor->GetActorLocation() + (coneTargetDistance * coneStartActor->GetActorForwardVector());

                    // Point the line toward the cone location
                    lineGuidanceActors[1]->SetActorRotation(UKismetMathLibrary::FindLookAtRotation(lineGuidanceActors[1]->GetActorLocation(), targetLocation));
                }

                if (currentGuidanceType == EGuidanceSpecificType::Type1 && root->bHiddenInGame) root->SetHiddenInGame(false);
                else if (currentGuidanceType == EGuidanceSpecificType::Type2 && !root->bHiddenInGame) root->SetHiddenInGame(true);
            }
            if (currentGuidanceMethod == EGuidanceMethod::CombineGuidance || currentGuidanceMethod == EGuidanceMethod::Vocal)
            {
                if (!isSoundPlaying)
                {
                    USoundBase* soundToPlay;

                    if (IsInsideACone(coneStartActor->GetActorLocation(), coneStartActor->GetActorForwardVector(), 2.0f, 20.0f, hapticConeAngle, mirror->MirrorReflectionMesh->GetComponentLocation()))
                    {
                        if (!seeToolTip)
                        {
                            isSoundPlaying = true;

                            soundToPlay = reminderSounds[5];
                            UGameplayStatics::PlaySound2D(GetWorld(), soundToPlay);
                            GetWorldTimerManager().SetTimer(timeHandler[0], resetSoundInstructionDelegate, soundToPlay->GetDuration() + SOUND_DELAY_TIME, false);
                        }
                        else if (angle > currentAngleThreshold)
                        {
                            isSoundPlaying = true;

                            soundToPlay = (reminderCounters[0]++ > MAX_SOUND_PLAY_TIME) ? reminderSounds[0] : reminderSounds[1];
                            UGameplayStatics::PlaySound2D(GetWorld(), soundToPlay);
                            GetWorldTimerManager().SetTimer(timeHandler[0], resetSoundInstructionDelegate, soundToPlay->GetDuration() + SOUND_DELAY_TIME, false);
                        }
                    }
                    else
                    {
                        isSoundPlaying = true;

                        soundToPlay = reminderSounds[4];
                        UGameplayStatics::PlaySound2D(GetWorld(), soundToPlay);
                        GetWorldTimerManager().SetTimer(timeHandler[0], resetSoundInstructionDelegate, soundToPlay->GetDuration() + SOUND_DELAY_TIME, false);
                    }
                }
            }
            if (currentGuidanceMethod == EGuidanceMethod::CombineGuidance || currentGuidanceMethod == EGuidanceMethod::Haptic)
            {
                if (!applyForce && !IsInsideACone(coneStartActor->GetActorLocation(), coneStartActor->GetActorForwardVector(), hapticSmallRadius, hapticBigRadius, hapticConeAngle, mirror->MirrorReflectionMesh->GetComponentLocation()))
                {
                    applyForce = true;
                }
                else if (applyForce && IsInsideACone(coneStartActor->GetActorLocation(), coneStartActor->GetActorForwardVector(), hapticSmallRadius + 0.5f, hapticBigRadius - 0.5f, hapticConeAngle - CONE_ANGLE_SUBTRACTOR, mirror->MirrorReflectionMesh->GetComponentLocation()))
                {
                    applyForce = false;

                    hapticForce = FVector(FMath::FRandRange(-.005f, .005f), FMath::FRandRange(-.005f, .005f), FMath::FRandRange(-.005f, .005f));
                    setDeviceForce(tool->DeviceIdMirror, hapticForce);
                }

                if (angle > currentAngleThreshold) // tooth is out view
                {
                    if (currentGuidanceType == EGuidanceSpecificType::Type1) // Hybrid
                    {
                        if (!shakeHaptic && FVector::Distance(targetLocation, mirror->MirrorReflectionMesh->GetComponentLocation()) < HAPTIC_DISTANCE_THRESHOLD / 4.0f)
                        {
                            shakeHaptic = true;
                        }
                        else if (shakeHaptic && FVector::Distance(targetLocation, mirror->MirrorReflectionMesh->GetComponentLocation()) > HAPTIC_DISTANCE_THRESHOLD)
                        {
                            shakeHaptic = false;
                        }

                        if (shakeHaptic)
                        {
                            hapticForce = FVector(FMath::FRandRange(-.25f, .25f), FMath::FRandRange(-.25f, .25f), FMath::FRandRange(-.25f, .25f));
                        }
                        else
                        {
                            FTransform hapticTransform = FTransform(
                                UKismetMathLibrary::FindLookAtRotation(mirror->MirrorReflectionMesh->GetComponentLocation(), targetLocation),
                                mirror->MirrorReflectionMesh->GetComponentLocation(),
                                FVector(1.0f)
                            );

                            hapticForce = UKismetMathLibrary::MakeRelativeTransform(hapticTransform, triggerTransform).GetRotation().GetForwardVector();
                            hapticForce.Normalize();

                            float magnitude = UKismetMathLibrary::FClamp(FVector::Dist(targetLocation, mirror->MirrorReflectionMesh->GetComponentLocation()) * HAPTIC_MOVEMENT_TYPE3_STIFFNESS, 0.0f, 1.0f);


                            hapticForce *= magnitude;

                        }
                    }

                    setDeviceForce(tool->DeviceIdMirror, FVector(hapticForce.X, -hapticForce.Y, hapticForce.Z));
                }
                else if (angle < currentAngleThreshold) // Tooth in the view
                {
                    if (IsInsideACone(coneStartActor->GetActorLocation(), coneStartActor->GetActorForwardVector(), hapticSmallRadius, hapticBigRadius, hapticConeAngle, mirror->MirrorReflectionMesh->GetComponentLocation()))
                    {
                        targetLocation = UKismetMathLibrary::VInterpTo(targetLocation, mirror->MirrorReflectionMesh->GetComponentLocation(), DeltaTime, 10.0f);
                    }

                    if (!seeToolTip)
                    {
                        FVector targetVisibilityLocation = toothTip->GetComponentLocation();
                        FVector blockerLocation = toolBlocker->GetComponentLocation();
                        float blockerRadius = toolBlocker->GetScaledSphereRadius() * 1.1f;
                        FVector mirrorLocation = mirror->MirrorReflectionMesh->GetComponentLocation();

                        targetLocation = FindRouteAroundSphere(targetVisibilityLocation, mirrorLocation, blockerLocation, blockerRadius);

                        FTransform hapticTransform = FTransform(
                            UKismetMathLibrary::FindLookAtRotation(mirror->MirrorReflectionMesh->GetComponentLocation(), targetLocation),
                            mirror->MirrorReflectionMesh->GetComponentLocation(),
                            FVector(1.0f)
                        );

                        hapticForce = UKismetMathLibrary::MakeRelativeTransform(hapticTransform, triggerTransform).GetRotation().GetForwardVector();
                        hapticForce.Normalize();

                        float magnitude = UKismetMathLibrary::FClamp(FVector::Dist(targetLocation, mirror->MirrorReflectionMesh->GetComponentLocation()) * HAPTIC_MOVEMENT_TYPE3_STIFFNESS, 0.0f, 1.0f);


                        hapticForce *= magnitude;

                        setDeviceForce(tool->DeviceIdMirror, hapticForce);
                    }
                    else
                    {
                        if (abs(hapticForce.Size()) > 0.015f)
                        {
                            hapticForce = FVector(FMath::FRandRange(-.005f, .005f), FMath::FRandRange(-.005f, .005f), FMath::FRandRange(-.005f, .005f));
                            setDeviceForce(tool->DeviceIdMirror, hapticForce);
                        }
                    }
                }
                debugActor->SetActorLocation(targetLocation);
            }
        }
        else
        {
            if (currentGuidanceMethod == EGuidanceMethod::CombineGuidance || currentGuidanceMethod == EGuidanceMethod::Visual)
            {
                if (coneMesh->IsVisible())
                {
                    coneMesh->SetVisibility(false);
                }

                float opacity;
                mirrorDynMAT->GetScalarParameterValue(TEXT("Opacity"), opacity);

                if (opacity > 0)
                {
                    opacity -= DeltaTime;
                    mirrorDynMAT->SetScalarParameterValue(TEXT("Opacity"), opacity);
                }

                lineDynMAT->GetScalarParameterValue(TEXT("Opacity"), opacity);
                if (opacity > 0)
                {
                    opacity -= DeltaTime;
                    lineDynMAT->SetScalarParameterValue(TEXT("Opacity"), opacity);
                }
            }
            if (currentGuidanceMethod == EGuidanceMethod::CombineGuidance || currentGuidanceMethod == EGuidanceMethod::Haptic)
            {
                if (!hapticForce.Equals(FVector(), 0.015f))
                {
                    hapticForce = FVector(FMath::FRandRange(-.005f, .005f), FMath::FRandRange(-.005f, .005f), FMath::FRandRange(-.005f, .005f));
                    setDeviceForce(tool->DeviceIdMirror, hapticForce);
                }
            }
        }

    }
    else if (triggerActor != nullptr && FVector::Distance(triggerActor->GetActorLocation(), mirror->MirrorReflectionMesh->GetComponentLocation()) < 4.0f)
    {
        float newTriggerYaw = UKismetMathLibrary::FindLookAtRotation(triggerTransform.GetLocation(), postureCheckerActor->GetActorLocation()).Yaw;
        triggerActor->SetActorRotation(FRotator(triggerActor->GetActorRotation().Pitch, newTriggerYaw, triggerActor->GetActorRotation().Roll));

        triggerTransform = triggerActor->GetActorTransform();
        targetLocation = mirror->MirrorReflectionMesh->GetComponentLocation();

        toothAnchorTransform = triggerActor->GetActorTransform();
        toothAnchorTransform.SetLocation(activeTooth->GetActorLocation());

        triggerActor = nullptr;

        initialRotation = GetActorRotation();


    }
}
// End -- Adjust the Ghost Mesh Behaviour Relative to Distance Between Ghost Mesh and Mirror Based on Guidance Method

// Start -- Save mirror location in regard to the tooth location
UKismetMathLibrary::MakeRelativeTransform(mirror->MirrorReflectionMesh->GetComponentTransform(), toothWorldRotationActor->GetActorTransform());
mirrorPositionLog.Append(
    mirror->MirrorReflectionMesh->GetComponentLocation().ToString() +
    "\t" + mirror->MirrorReflectionMesh->GetComponentRotation().ToString() + "\n"
);
// End -- Save mirror location in regard to the tooth location

```

}

float AGuidanceActor::FindSignedAngleBetween2DVectors(FVector2D a, FVector2D b)

{

return UKismetMathLibrary::Atan2((a.X * b.Y) - (a.Y * b.X), (a.X * b.X) + (a.Y * b.Y));

}

FVector AGuidanceActor::GetReflectionVector(FVector sorceLocation, FVector reflectionPoint, FVector reflectionPointNormal)

{

FVector p_0 = sorceLocation; // start point FVector p_r = reflectionPoint; // reflection point FVector n = reflectionPointNormal; // normal of the reflection point

FVector i = p_r - p_0; // direction from start point to reflection point
i.Normalize();

FVector r = i - (2.0f * (FVector::DotProduct(i, n) * n)); // reflection ray
r.Normalize();

return r;

}

void AGuidanceActor::ChangeCurrentGuidance()

{

switch (currentGuidanceMethod) {

case EGuidanceMethod::Visual: root->SetHiddenInGame(true);

```
   lineGuidanceActors[0]->SetActorHiddenInGame(true);
    lineGuidanceActors[1]->SetActorHiddenInGame(true);

    coneMesh->SetVisibility(false);

    isSoundPlaying = true;
    UGameplayStatics::PlaySound2D(GetWorld(), instructionIntroSound);
    GetWorldTimerManager().SetTimer(timeHandler[0], resetSoundInstructionDelegate, instructionIntroSound->GetDuration() + SOUND_DELAY_TIME, false);

    currentGuidanceMethod = EGuidanceMethod::Vocal;
    break;
case EGuidanceMethod::Vocal:
    root->SetHiddenInGame(true);

    lineGuidanceActors[0]->SetActorHiddenInGame(true);
    lineGuidanceActors[1]->SetActorHiddenInGame(true);

    coneMesh->SetVisibility(false);

    currentGuidanceMethod = EGuidanceMethod::Haptic;
    break;
case EGuidanceMethod::Haptic:
    root->SetHiddenInGame(false);

    lineGuidanceActors[0]->SetActorHiddenInGame(true);
    lineGuidanceActors[1]->SetActorHiddenInGame(false);

    coneMesh->SetVisibility(true);

    isSoundPlaying = true;
    UGameplayStatics::PlaySound2D(GetWorld(), instructionIntroSound);
    GetWorldTimerManager().SetTimer(timeHandler[0], resetSoundInstructionDelegate, instructionIntroSound->GetDuration() + SOUND_DELAY_TIME, false);

    currentGuidanceMethod = EGuidanceMethod::CombineGuidance;
    break;
case EGuidanceMethod::CombineGuidance:
    root->SetHiddenInGame(true);

    lineGuidanceActors[0]->SetActorHiddenInGame(true);
    lineGuidanceActors[1]->SetActorHiddenInGame(true);

    coneMesh->SetVisibility(false);

    currentGuidanceMethod = EGuidanceMethod::NoGuidance;
    break;
case EGuidanceMethod::NoGuidance:
    root->SetHiddenInGame(false);

    lineGuidanceActors[0]->SetActorHiddenInGame(true);
    lineGuidanceActors[1]->SetActorHiddenInGame(false);

    coneMesh->SetVisibility(true);

    currentGuidanceMethod = EGuidanceMethod::Visual;
    break;
default:
    break;
}
```

}

FVector AGuidanceActor::FindRouteAroundSphere(const FVector& PointA, const FVector& PointD, const FVector& SphereCenter, float SphereRadius)

{

// Step 1: Check if line AD intersects the sphere

FVector LineDirection = PointD - PointA; float LineLength = LineDirection.Size();

```
if (LineLength < KINDA_SMALL_NUMBER)
{
    // A and D are the same point
    return PointD;
}

// Find closest point on line segment AD to sphere center
FVector ToSphere = SphereCenter - PointA;
float T = FVector::DotProduct(ToSphere, LineDirection) / LineDirection.SizeSquared();
T = FMath::Clamp(T, 0.0f, 1.0f);

FVector ClosestPoint = PointA + T * LineDirection;
float DistanceToSphere = FVector::Dist(ClosestPoint, SphereCenter);

// If no intersection, return original D
if (DistanceToSphere > SphereRadius)
{
    return PointD;
}

// Step 2: Calculate tangent from A to sphere
FVector AB = SphereCenter - PointA;
float DistanceAB = AB.Size();

// Check if A is inside the sphere
if (DistanceAB <= SphereRadius)
{
    UE_LOG(LogTemp, Warning, TEXT("Point A is inside or on the sphere. Cannot calculate tangent."));
    return PointD;
}

// Calculate tangent length using Pythagorean theorem
// tangentLength^2 + radius^2 = distanceAB^2
float TangentLength = FMath::Sqrt(DistanceAB * DistanceAB - SphereRadius * SphereRadius);

// Find the tangent point T
// The angle alpha between AB and AT
float CosAlpha = TangentLength / DistanceAB;
float SinAlpha = SphereRadius / DistanceAB;

// Direction from A to B
FVector DirectionAB = AB.GetSafeNormal();

// We need to find which tangent to use (there are 2 tangents from A to the sphere)
// Choose the one closest to the original direction AD
FVector DirectionAD = LineDirection.GetSafeNormal();

// Get a perpendicular vector to AB in the plane containing A, B, and D
FVector Perpendicular = GetPerpendicularInPlane(DirectionAB, DirectionAD);

// Calculate two possible tangent directions
FVector TangentDir1 = CosAlpha * DirectionAB + SinAlpha * Perpendicular;
FVector TangentDir2 = CosAlpha * DirectionAB - SinAlpha * Perpendicular;

// Choose the tangent direction closer to the original AD direction
FVector TangentDirection;
if (FVector::DotProduct(TangentDir1, DirectionAD) > FVector::DotProduct(TangentDir2, DirectionAD))
{
    TangentDirection = TangentDir1.GetSafeNormal();
}
else
{
    TangentDirection = TangentDir2.GetSafeNormal();
}

FVector DPrime = PointA + LineLength * TangentDirection;

return DPrime;
```

}

FVector AGuidanceActor::GetPerpendicularInPlane(const FVector& Primary, const FVector& Reference)

{

// Project reference onto primary

FVector Projection = FVector::DotProduct(Primary, Reference) * Primary;

```
// Perpendicular component
FVector Perpendicular = Reference - Projection;

// If perpendicular is too small, choose arbitrary perpendicular
if (Perpendicular.Size() < 0.001f)
{
    // Choose an arbitrary perpendicular direction
    if (FMath::Abs(Primary.X) < 0.9f)
    {
        Perpendicular = FVector(1, 0, 0);
    }
    else
    {
        Perpendicular = FVector(0, 1, 0);
    }
    Perpendicular = FVector::CrossProduct(Primary, Perpendicular);
}

return Perpendicular.GetSafeNormal();
```

}

bool AGuidanceActor::IsInsideACone(FVector origin, FVector direction, float radius1, float radius2, float coneAngle, FVector point)

{

float smallRadius, largeRadius;

if (radius1 < radius2)

{

smallRadius = radius1;

largeRadius = radius2;

}

else

{

smallRadius = radius2;

largeRadius = radius1;

}

FVector originToPointDir = point - origin; originToPointDir.Normalize();

float angle = UKismetMathLibrary::DegAcos(FVector::DotProduct(direction, originToPointDir));
float distance = FVector::Dist(origin, point);

if ((smallRadius < distance && distance < largeRadius) && angle < coneAngle / 2.0f)	return true;

return false;

}
