# Supplementary material for: Virtual Reality–Based Mirror Guidance for Indirect Vision Training in Minimally Invasive Endodontic Access Cavity Preparation Among Dental Students: Randomized Controlled Crossover Trial
Source: JMIR Serious Games. 2026 Oct 1;14:e100884. doi: 10.2196/100884 (PMC13673179; doi:10.2196/100884)
Supplement: Supplementary file 4 [file games_v14i1e100884_app4.docx]

**VR-based mirror guidance system screenshots**

**The Compiled Version of the Software**

The compiled version of the software can be found at the bottom of the following [webpage](https://cgvr.cs.uni-bremen.de/research/dentalsim/).

**Mirror Guidance Condition**

|  |  |  |
| --- | --- | --- |
| Condition A  Mirror is Outside the Optimal Area | **Condition B**  Mirror is Incorrectly Oriented | **Condition C**  Tooth is Occluded |

The figures show the visualization of each mirror guidance condition.

**Verbal Guidance**

This guidance method could not be demonstrated using figures. The video demonstration of the guidance method can be found [here](https://cgvr.cs.uni-bremen.de/research/dentalsim/).

**Visual Guidance**

|  |  |  |
| --- | --- | --- |
| Condition A  Mirror is Outside the Optimal Area | **Condition B**  Mirror is Incorrectly Oriented | **Condition C**  Tooth is Occluded |

The figures show the visualization of each visual guidance component regarding each mirror guidance condition.

The video demonstration of the guidance method can be found [here](https://cgvr.cs.uni-bremen.de/research/dentalsim/).

**Haptic Guidance**

This guidance method could not be demonstrated using either figures or videos.
